# The temporal window of individuation limits visual capacity

**DOI:** 10.3389/fpsyg.2014.00952

**Published:** 2014-08-27

**Authors:** Andreas Wutz, David Melcher

**Affiliations:** Active Perception Laboratory, Center for Mind/Brain Sciences, University of TrentoRovereto Italy

**Keywords:** visual capacity, temporal window, oscillatory phase synchrony, individuation, integration

## Abstract

One of the main tasks of vision is to individuate and recognize specific objects. Unlike the detection of basic features, object individuation is strictly limited in capacity. Previous studies of capacity, in terms of subitizing ranges or visual working memory, have emphasized spatial limits in the number of objects that can be apprehended simultaneously. Here, we present psychophysical and electrophysiological evidence that capacity limits depend instead on time. Contrary to what is commonly assumed, subitizing, the reading-out a small set of individual objects, is not an instantaneous process. Instead, individuation capacity increases in steps within the lifetime of visual persistence of the stimulus, suggesting that visual capacity limitations arise as a result of the narrow window of feedforward processing. We characterize this temporal window as coordinating individuation and integration of sensory information over a brief interval of around 100 ms. Neural signatures of integration windows are revealed in reset alpha oscillations shortly after stimulus onset within generators in parietal areas. Our findings suggest that short-lived alpha phase synchronization (≈1 cycle) is key for individuation and integration of visual transients on rapid time scales (<100 ms). Within this time frame intermediate-level vision provides an equilibrium between the competing needs to individuate invariant objects, integrate information about those objects over time, and remain sensitive to dynamic changes in sensory input. We discuss theoretical and practical implications of temporal windows in visual processing, how they create a fundamental capacity limit, and their role in constraining the real-time dynamics of visual processing.

## INTRODUCTION – VIRTUAL CONTINUITY AND STABILITY OF PERCEPTUAL SPACE AND TIME

The perception system is faced with the task of transforming continuous sensory input into discrete objects and events. It is critical for survival that the perceptual system is sensitive and quickly responsive to changes in the input over time in order, for example, to detect and interpret signals regarding object or self-motion. However, a primary goal of perceptual systems is also to uncover stability in the identity and location of spatiotemporal objects and to integrate information over extended periods of time in order to understand complex phenomena such as biological motion (Neri et al., 1998) or events (Hasson et al., 2008; Lerner et al., 2011; Zacks and Magliano, 2011). Information must be integrated over time to recover the regularities in the world and to use this perception of order to make predictions about the near future (Nastase et al., 2014). Thus, vision in real-time requires a balance combining information over time (in order to integrate motion signals or to keep track of the same spatiotemporal object) and sensitivity to new information.

A simple example of this challenge for a perceptual system is the task of crossing a busy street. Perceiving and predicting the motion of vehicles requires combining information over 100s of milliseconds or even seconds, often including the combination of motion information across occlusion or changes in retinal position caused by eye movements. On the one hand, combining information over a longer time period would likely lead to the best possible estimate of all of the features of the oncoming cars. Nonetheless, the visual system must also provide a good enough estimate of the current location of each vehicle in order to support action. Thus, the perceptual system must optimally balance the competing needs of speed and information: more time yields better information but slows down the ability of the organism to react rapidly to the current state of affairs. It seems likely that the brain provides a compromise by utilizing a hierarchy of different temporal integration windows (Pöppel, 1997, 2009; Hasson et al., 2008; Melcher and Colby, 2008; Holcombe, 2009; Lerner et al., 2011; Masquelier et al., 2011) and by alternating periods of feedforward sampling of new information with feedback/re-entrant processes (Di Lollo et al., 2000; Lamme and Roelfsema, 2000) that create a perceptual synthesis of the disparate sensory information into coherent, stable spatio-temporal entities like objects. Indeed, converging evidence suggests that temporal limits on visual processing can be broadly divided into two groups of perceptual mechanisms (Holcombe, 2009). A fast group comprises processes of feedforward feature detection and works on the scale of some 10s of milliseconds. The second group of visual mechanisms is much slower, taking more than at least 100 ms and operates on more high-level properties, like objects that have been selected and individuated.

Here we consider evidence regarding how the temporal window of object individuation might bridge the gap between fast feedforward sampling of information and slower object-based computations. We start with a selective review of the relevant literature on object individuation, its capacity limits and temporal limits in visual perception. Then, we describe a methodology to experimentally reduce the effective visual persistence of a visual display in order to more closely map out the time course of object individuation processes. We review our recent behavioral studies using this method to show the unfolding of object individuation and working memory over time. Then we present and discuss magnetencephalography (MEG) evidence regarding the neural correlates of this process, including the possibility that neural synchronization patterns can provide useful information about the nature of integration and individuation. Finally, we discuss the implications of these findings for capacity limits in visual cognition, their relationship with natural vision and oscillatory brain dynamics, and point out some open questions and directions for future research.

## INDIVIDUATION MEASURES VISUO-SPATIAL OBJECT PROCESSING

### INDIVIDUATION: AN INTERMEDIATE STEP BETWEEN SAMPLING FEATURES AND OBJECTS

Although sensory information seems to extend continuously into perceptual space and time, the content of cognitive operations consists of coherent scenes containing a limited number of discrete and invariant objects in any particular instance (Treisman and Gelade, 1980; Tipper et al., 1990; Kahneman et al., 1992; Baylis and Driver, 1993; Scholl et al., 2001). Such parsing of the sensory environment into elemental perceptual units (Spelke, 1988) provides a link between sensation and cognition that couples perception to the external world, free from an infinite regress of referring to semantic categories (Pylyshyn, 2001). Reading-out objects from feedforward sensory input is called individuation and involves selecting features from a crowded scene, binding them into a unitary spatiotemporal entity and segregating this perceptual unit from other individuals in the image (Treisman and Gelade, 1980; Xu and Chun, 2009). The output of this intermediate-level visual analysis is a stable object-based reference frame in which the different features of a specific location in the scene can be bound together.

Object representations at this stage are suggested to be coarse and contain only minimal feature information. In fact such individual entities do not necessarily provide information about object identity, but can be regarded as a spatio-temporal placeholder of the object in focus until feedback processes fill in content. Several theoretical, psychophysical and neuroimaging studies have emphasized the computational importance and necessity of such incremental object representations in intermediate-level vision, with these entities described as visual indexes (Pylyshyn, 1989), proto objects (Rensink, 2000), or object-files (Kahneman et al., 1992; Xu and Chun, 2009). In its essence an object can be defined as an entity whose recent spatio-temporal history can be reviewed and therefore still can be referred to as the same entity despite of changes in its location over time (Kahneman et al., 1992).

Individuation is an intermediate step in object processing between bottom-up feature detection and the recognition of stable and coherent objects. Object representations at this level of processing are commonly measured with an enumeration task that solely requires knowing whether an object is an individual rather than its identity (which is usually measured with change detection and interpreted as the content of visual working memory). In this review we will map the temporal dynamics of visual object processing as a cascade from (a) sampling a visual signal over (b) a temporal window of ca. 100 ms duration during which a scene is segmented and individuated into (c) stable object-based representations. Our main result characterizes a brief time window of persisting sensory information after stimulus onset that limits object individuation and accounts for capacity limits in visual object processing.

### CAPACITY LIMITS IN INDIVIDUATION AND VISUAL MEMORY

Although human cognition is remarkably powerful, its online workspace, working memory, appears to be highly limited in the number of informational units it processes (Miller, 1956; Luck and Vogel, 1997; Cowan, 2000). It is interesting to note that this capacity is linked to cognitive abilities in general. For example, inter-individual variability in measures of fluid intelligence and capacity estimates are highly correlated (Engle et al., 1999; Cowan et al., 2005; Fukuda et al., 2010b) and reduced capacity is often found in patients with neuropsychiatric disorders (Karatekin and Asarnow, 1998; Lee et al., 2010).

**FIGURE 1:**
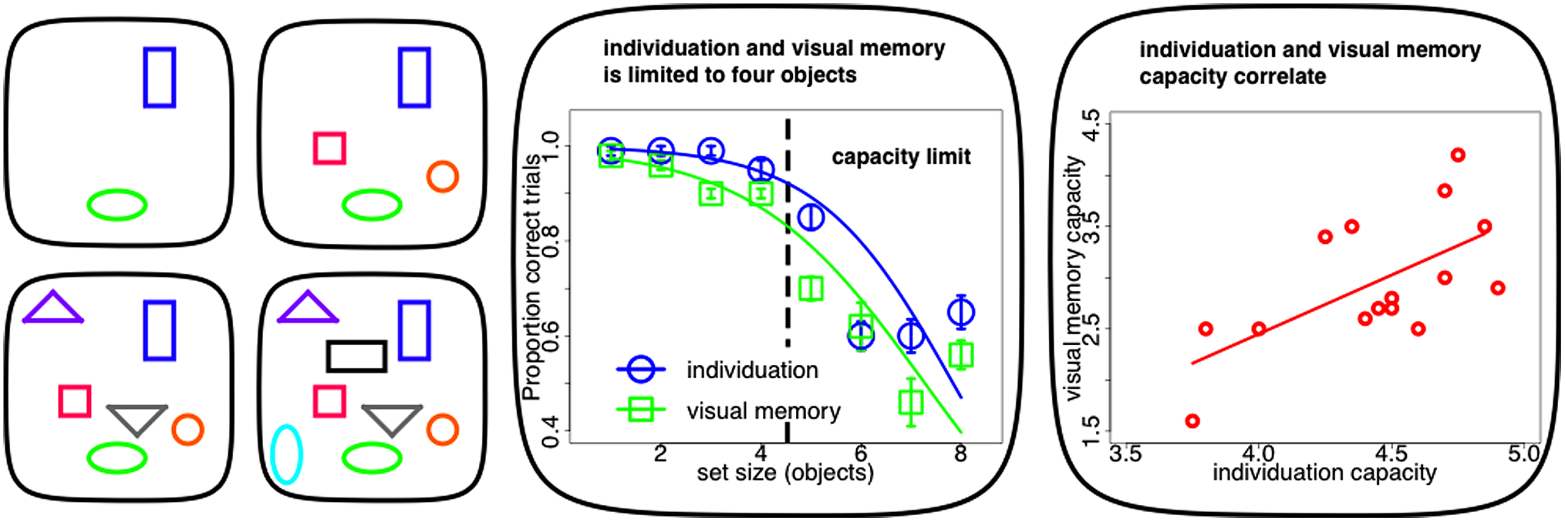
**Capacity limits in visuo-spatial object processing. Left panel:** typical stimuli within experiments on visuo-spatial object processing (individuation, visual memory). Individuation for up to four objects (upper panels) is accurate and fast. Visuo-spatial object processing above this limit requires successive perceptual steps (counting, lower panels). **Middle panel:** visuo-spatial object processing (individuation, visual memory) as a function of set-size. Both tasks show a limit of up to four objects. The inflection point of the sigmoid curve fit to the psychophysical data can be used to estimate individual capacity limits. **Right panel:** single-subject correlation between individuation and visual memory capacity. Limits in visuo-spatial object processing correlate across subjects and individuation usually exceed visual memory limits (Figures adapted with permission from Piazza et al., 2011).

Recent evidence suggests that two distinct mechanisms, object individuation and identification, work together in creating these visual object capacity limitations (Xu and Chun, 2009). Individuation appears to be the initial bottleneck in visual object processing from an unlimited in capacity, but fragile, purely bottom-up and in parallel computed sensory representation (iconic memory: Sperling, 1960, 1963; Neisser, 1967) to such a capacity limited, durable and cognitively structured visual store (visual short-term memory: Sperling, 1960, 1963; Phillips and Baddeley, 1971). A subset of these individuated objects are elaborated subsequently during object identification. It is at this stage that identity information becomes available to the observer and the content of the object can be consolidated into durable and reportable representations in visual working memory (Xu and Chun, 2009). As individuation precedes identification, the capacity of the latter has its upper bound in the limit of the former (Melcher and Piazza, 2011; Dempere-Marco et al., 2012; **Figure 1** middle panel). In fact on a single-subject level, estimates of individuation capacity commonly exceed visual memory limits and the two measures tend to be highly correlated (Piazza et al., 2011; **Figure 1** right panel).

It has long been noted that individuation is limited in capacity: we can quickly and effortlessly perceive that there are exactly two items but not that there are exactly eight items (Jevons, 1871; compare **Figure 1** left panel upper row with lower row). Enumeration is equally quick, accurate and effortless within a narrow range of one to four objects. Such small numbers of items are supposedly simultaneously apprehended by a qualitatively distinct mechanism known as “subitizing” (Kaufman et al., 1949). Performance for set-sizes exceeding this range, as measured by reaction time and accuracy, deteriorates with every additional item to be enumerated (**Figure 1** middle panel). This suggests that visual object capacity limits are grounded in this “subitizing” phenomenon and that visual processing beyond this limit has to rely on imprecise estimation or serial and time-consuming counting that requires successive perceptual steps. In contrast, “subitizing” is thought to measure visuo-spatial object processing within one single feedforward processing iteration (for review, see Melcher and Piazza, 2011; Piazza et al., 2011).

### THEORIES ABOUT OBJECT PROCESSING CAPACITY LIMITS

In light of its importance for cognitive and perceptual functioning, the search for the root of this capacity limitation is fundamental to the study of visual cognition. There are a number of competing theories for why “subitizing,” and individuation in general, is limited to sets of only about three or four items (for review, see Piazza et al., 2011). These theories start with the idea that capacity measures the number of objects individuated “immediately” (Kaufman et al., 1949), as reflected in the root of the word “subitizing” (*subitus*). This capacity is characterized in terms of spatial metaphors such as an index, pointer, or slots. Capacity is thus typically thought of as a limit in spatial resolution, rather than temporal limits. Because of the apparent automaticity and immediateness of processing, several theories assumed an *ad hoc*, direct and continuous indexing between external coordinates and object-files (Pylyshyn, 1989), like focal slots waiting to be filled in with content (Luck and Vogel, 1997; Fukuda et al., 2010a). Since performance tends to deteriorate after around four items (although this does depend on individuals and task), it was proposed that there were four indexes or slots.

Starting with the idea that subitizing is an all-or-none, uniform process might, however, neglect the possibility that capacity is related to the temporal period during which individuation occurs. Individuation is a computationally complex task. Ullman (1984) has characterized vision in terms of serial tasks that involves indexing of salient items, marking previously indexed locations and multiple shifts of the processing focus. In fact, execution of such complex coding in real-time would seem likely to require the implementation of a specialized routine set-up as a series of elemental operations (Roelfsema et al., 2000). As reviewed in the following section, temporal aspects of visual perception have been studied extensively and show that visual processing is not “immediate” (Kaufman et al., 1949) but always occurs over time. This raises the question of whether these temporal factors, rather than or in addition to spatial factors, might underlie capacity limits.

In terms of time, object individuation is a process that must, as described above, balance between the need for speed and the aim of integrating information over time about salient objects in order to recognize, remember and respond to their properties. This trade-off is apparent in the case of computer vision systems for robotics, in which an exact, metric representation of the environment is computationally expensive and typically too slow to guide behavior in real-time. Computer systems used to drive cars, for example, do not represent in detail the entire visual scene (Bertozzi et al., 2000) because such a complete, metric model cannot be updated in real-time. In the case of the human visual system, one strategy to deal with this trade-off is to individuate and integrate information about a small number of potentially important items within each perceptual cycle.

## TEMPORAL RESOLUTION OF VISUO-SPATIAL OBJECT PROCESSING

### VISUO-TEMPORAL LIMITS BETWEEN FEATURE DETECTION AND OBJECT-BASED COMPUTATIONS

Temporal resolution refers to the precision of a measurement with respect to time. Estimates of the temporal resolution of vision come from a variety of different tasks but can be divided into two groups of temporal limits: a fast group that operates on the order of 10s of milliseconds and a slower group of visual mechanisms taking more than 100 ms (Holcombe, 2009). The fast temporal limits are usually explained by temporal integration of low-level visual features (like in the case of flicker fusion or integration masking; Crozier and Wolf, 1941; Kietzman and Sutton, 1968; Scheerer, 1973a,b; Di Lollo and Wilson, 1978; Coltheart, 1980; Enns and Di Lollo, 2000; Breitmeyer and Öğmen, 2006). In contrast, slower temporal limits are usually associated with high-level processing in an object-based frame of reference like in the case of feature conjunctions across space (color-shape: Holcombe and Cavanagh, 2001; or orientation-location: Motoyoshi and Nishida, 2001) or consolidation of objects in visual working memory (Gegenfurtner and Sperling, 1993; Vogel et al., 2006). Unlike the temporal blurring of basic image features, temporal processing limits for this slower group have been suggested to depend on selective attention (Holcombe, 2009). Together these two groups of processes act in concert to create a coherent perceptual impression in time.

Here, we try to combine these two frameworks, temporal resolution and attentional selection. As reviewed above, object individuation appears to be the basic set-up process for object-based representations, introducing selectivity in processing individual properties of a scene. Consistent with this idea recent evidence suggests that “subitizing” and individuation in general, rather than being a pre-attentive indexing mechanism (Trick and Pylyshyn, 1994), requires selective attention (Egeth et al., 2008; Olivers and Watson, 2008; Railo et al., 2008). We show that individuation is limited by temporal integration of sensory information over time and how visual capacity limits arise naturally as a consequence of this integration window. We argue that intermediate-level vision bridges the gap between fast feature detection and slower object-based computations, and that this depends on a temporal integration window that is used to structure and stabilize individual perceptual elements within a sampled sensory image.

### TEMPORAL INTEGRATION OF SENSORY PERSISTENCE

Following stimulus onset a briefly presented visual display persists perceptually for a limited temporal window of 80–120 ms (Haber and Standing, 1970; Coltheart, 1980; Di Lollo, 1980). This persisting window acts like a low-pass filter on dynamic aspects of real-time vision, limiting the temporal resolution of perceiving each single visual event. When a second stimulus is presented in rapid succession to a first stimulus, the associated features of both stimulus onsets are partly integrated into a single percept. Such short-lived sensory integration intervals have been described to influence visual perception (Scheerer, 1973a; Enns and Di Lollo, 2000; Breitmeyer and Öğmen, 2006), visual memory (Di Lollo, 1980), and rapid perceptual decision-making (Scharnowski et al., 2009; Rüter et al., 2012).

**FIGURE 2:**
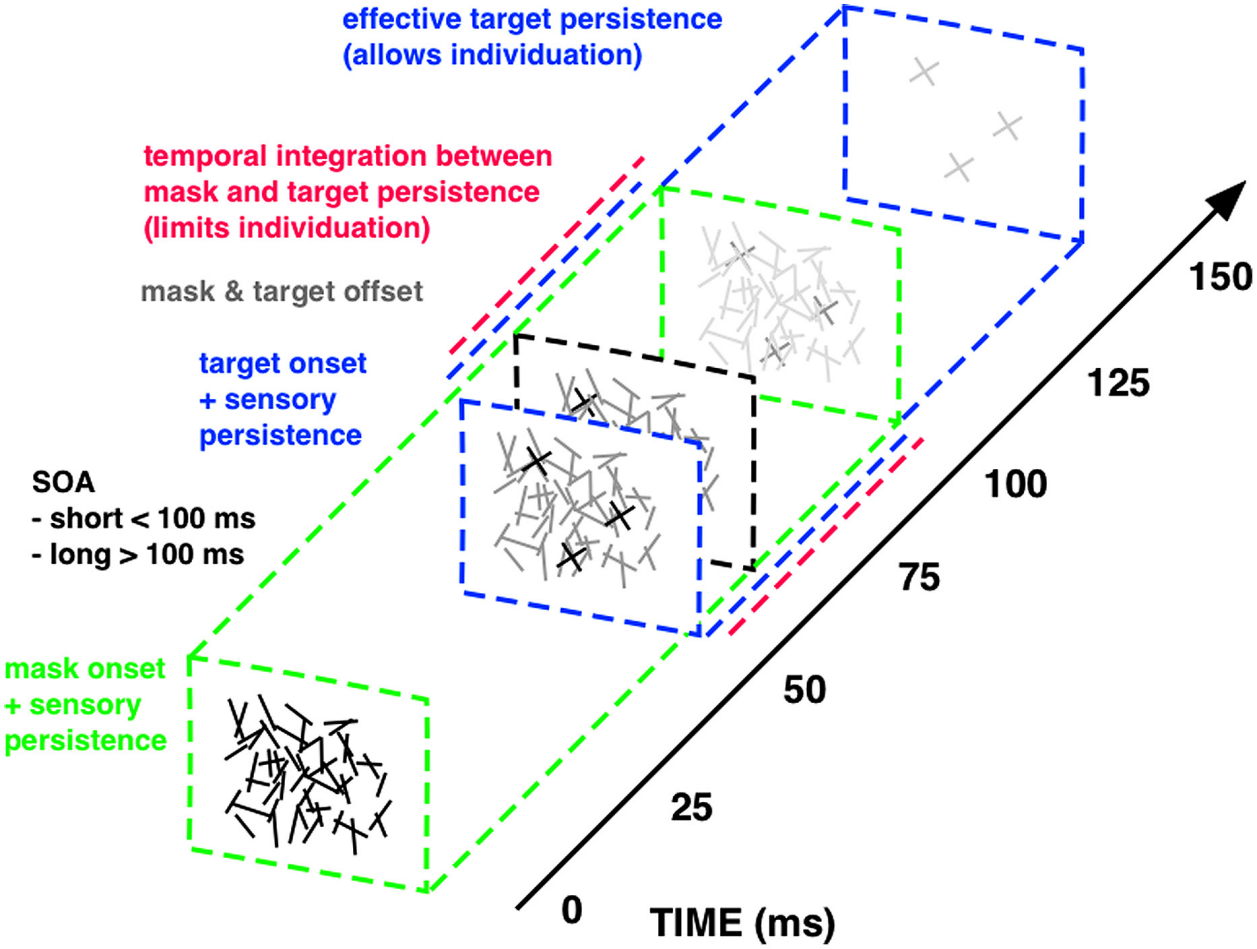
**Integration masking sequence.** The visual stimuli within this integration masking sequence, a random-line noise mask and the target elements (“X”), are rendered physically indistinguishable (i.e., equal luminance, equal mean line length and width, random spatial position of lines), enforcing integration of physical features via mask-target similarity (Blalock, 2013). Mask and target events also offset together at the same time (gray square at about 60 ms on above scale), so that temporal onset asynchrony between visual stimuli (SOA; 50 ms above) constitutes the only physical difference between mask (green square at 0 ms) and target events (blue square at 50 ms). Temporal integration of mask and target features (red, dashed line) occurs for SOAs shorter than around 100 ms, since masking triggered at mask onset continues for this quasi-constant period of sensory persistence (green, dashed line). With longer SOAs the sensory trace triggered at target onset (blue, dashed line) successively segregates from masking persistence and the therein-contained target information can be read-out for an increasingly longer interval. The maximum time window available for target read-out spans the entire effective target persistence (i.e., without integration from preceding masking persistence) of around 100 ms (see Wutz et al., 2012, 2014; Wutz and Melcher, 2013 for details on the masking sequence).

Important insights into the temporal dynamics of sensory integration have been achieved through the study of visual masking: the reduction of the visibility of one stimulus, called the target, by another stimulus shown before and/or after it, called the mask (Enns and Di Lollo, 2000; Breitmeyer and Öğmen, 2006). It is classically explained in terms of a two-factor theory: integration and interruption masking (Scheerer, 1973a,b). Interruption masking limits more high-level feedback processing after perceptual analysis of the target has largely finished. Integration masking, however, results from short-lived temporal collapsing of feedforward sensory signals, as a consequence of the imprecise temporal resolution of the visual system. Integration of sensory persistence between rapid successive stimuli reduces the time to access the sensory trace of each single stimulus. Hence integration masking degrades visual performance by fractionating the sensory persistence of the target display and limiting its effective presentation time. Integration masking is very effectively implemented with a specific forward masking technique that makes it possible to quantitatively change the duration of sensory persistence and the degree of temporal integration by varying the onset asynchrony between the first and second display (Di Lollo, 1980; Wutz et al., 2012; **Figure 2**).

**FIGURE 3:**
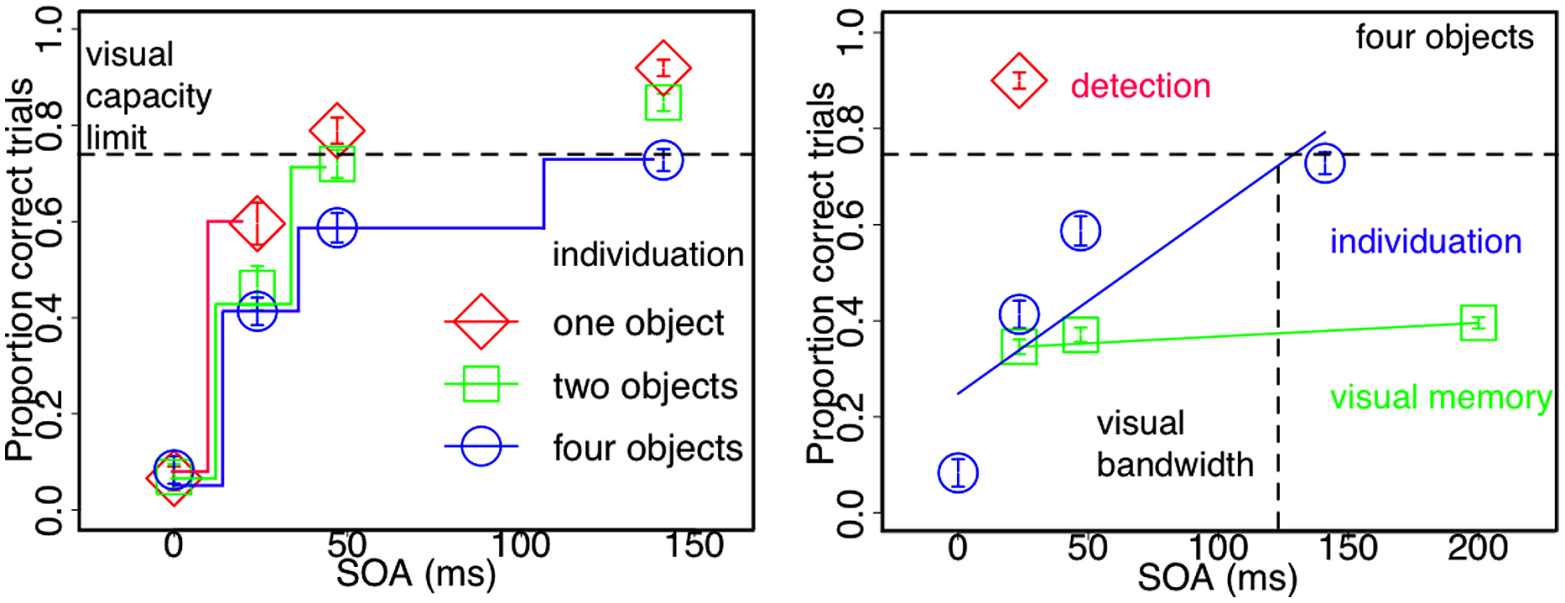
**Visuo-spatial object processing under conditions of integration masking. Leftpanel:** enumeration performance for one, two, and four objects as a function of stimulus onset a synchrony (SOA). Individuation capacity increases in steps as a function of SOA and hence less integration masking. One object can be individuated after 25 ms, two objects require 50 ms and the full-set of four objects (the average visuo-spatial capacity limit) are only stabilized within the entire life time of sensory persistence (100 ms). **Rightpanel:** detection is faster and visual memory slower than individuation. The onset of four visual stimuli can be reliably detected with as little as 25 ms between mask and target onsets. Individuation of four objects, however, increases in steps for up to 100 ms as a function of SOA. Visual memory for four objects that requires identity integration with individuated object-files remains stable and low across SOAs (Figures adapted with permission from Wutz et al., 2012 and Wutz and Melcher, 2013).

Mask and target elements share the same physical properties, in order to equate stimulus energy from both visual events. The only physical difference between mask and target constitutes their temporal onset asynchrony. Temporal integration of mask and target features occurs for stimulus onset asynchronies (SOAs) shorter than around 100 ms, because of smearing of sensory persistence triggered at each onset. For SOAs exceeding this critical time frame, mask and target persistence segregate in time and the sensory trace of the target display can be read-out. In this way, varying the SOA within this integration masking sequence controls the effective presentation time of a visual display by fractionating its sensory trace. We designed this technique to map the temporal dynamics of successive perceptual processes involved in object processing with identical visual stimuli only varying task demands: from basic detection to subsequent individuation and finally identification and consolidation of objects in visual working memory (**Figure 3**).

## INTEGRATION WINDOWS LIMIT INDIVIDUATION CAPACITY

### INDIVIDUATION CAPACITY INCREASES UNIT BY UNIT WITHIN THE SENSORY WINDOW

Individuation stabilizes visual perception by computing objects. This process is thought to operate within a single glance and is strictly limited in capacity to a small set of around four objects. We tested whether visual object capacity is indeed reached at the very moment a stimulus enters the visual field or instead accumulates with longer viewing time by fractionating a single glance into smaller units. We used an integration masking paradigm (see **Figure 2**) in order to vary the time to access the sensory trace of the to be individuated items and measured individuation performance for different set-sizes. Contrary to what is commonly found in “subitizing” tasks, which has consistently shown highly accurate performance up to around four objects across a wide range of studies (see **Figure 1**), fractionating the sensory persistence of the stimulus with integration masking dramatically reduces individuation capacity. This suggests that reading-out a small set of individual and stable objects is not an instantaneous process (**Figure 3**) but rather evolves over time.

Individuation capacity increases in steps within the lifetime of sensory persistence of the stimulus (**Figure 3**; Wutz et al., 2012). Within integration masking, SOA between mask and target directly reflects effective target persistence and time to read-out individual objects. Temporal integration of visual signals is complete and target information is completely inaccessible if there is common stimulus onset (SOA = 0 ms). With increasing SOA, visual signals segregate in time and the read-out of each single sensory trace increases correspondingly. The slopes of individuation across read-out time, however, co-vary with the number of individual objects to be processed. Whereas one object is sufficiently stable within 25 ms, two objects require 50 ms to be individuated. Individuation capacity for four objects, which is the average visuo-spatial capacity limit (**Figure 1**), is asymptotically reached after around 100 ms (**Figure 3** left panel; Wutz et al., 2012). Limiting the effective presentation time with integration masking reveals that processing speed and object capacity interact, rather than a uniform individuation improvement across the “subitizing range” with less temporal limitations. Consistent with this result, interactions between perceptual speed and object selection have also been reported for multiple object tracking (Holcombe and Chen, 2013).

Incremental individuation of objects within a stimulus’ sensory persistence suggests that this temporary integration buffer is functionally critical for object processing. Such an integration interval might reflect the need to equilibrate read-out of invariant and stable perceptual form and almost simultaneously integrate changes in sensory input into a continuous stream of visual impressions. Sensory images that remain stationary within the first 100 ms after sampling are successively segmented and structured into objects within its sensory persistence. Consequently, visuo-spatial object capacity limitations arise as a result of the narrow integration window bandwidth (**Figure 3**).

The speed of stable information accrual, however, is particularly crucial in case of fast changes in the sampled sensory image (<100 ms). When the sensory signal changes faster than the integration window (<100 ms; change, motion, short SOA masking sequence) individuation capacity is reduced as a function of the rate of sensory change, stabilizing only a subset of objects. This drop in visuo-spatial object processing with higher temporal processing demands balances the needs for perceptual stability in space and continuity in time. One object can already be stabilized in some 10s of milliseconds. In this way at least one object can be selected and further tracked for speeds drawing near the upper temporal limit of visual processing (Kietzman and Sutton, 1968). Structuring an entire scene into multiple objects, however, requires processing over an interval of around 100 ms. We argue that vision uses the time window of sensory persistence following stimulus onset to balance the opposing needs of individuating stable objects and maintaining the temporal resolution necessary to track rapidly changing events.

### SAMPLING FEATURES IS FASTER, WHILE VISUAL MEMORY IS SLOWER THAN INDIVIDUATION

Temporal buffering of input signals does not necessarily imply that sampling of new information is inhibited completely within this integration interval. In fact, merely detecting a second event requires as little as 25 ms between event onsets (**Figure 3** right panel). Despite this remarkable processing speed, the informational content of such fast feedforward sampling is considered to be virtually unlimited in capacity (Wundt, 1899; Sperling, 1960) and can already involve higher-level visual areas, allowing for rapid scene categorization of natural images (Thorpe et al., 1996; Li et al., 2002), basic image grouping (Field et al., 1993; Roelfsema et al., 2000), visual analysis of scene semantics (but not scene syntax, Vö and Wolfe, 2012; Võ and Wolfe, 2013) or computation of global summary statistics of the raw sensory image. For example, the average size of a set of objects can be computed even when the display changes continuously (Albrecht and Scholl, 2010). Thus certain global properties of the sensory image can be read-out during fast sampling, serving as a layout for visual analysis (“the gist”; Rensink, 2000).

Without translation into a perceptually invariant and stable representation, however, information about individual elements within the sensory image is easily over-written by subsequent input (Wundt, 1899; Sperling, 1960, 1963; Breitmeyer and Öğmen, 2006). Hence, selectivity in spatio-temporal processing does not arise from a failure to sample the sensory image, but reflects subsequent structuring and stabilization of individual perceptual elements (Wutz and Melcher, 2013). Accordingly, individuation (but not basic bottom-up detection) of multiple perceptual elements evokes a set-size specific modulation of the N2pc EEG-component that is commonly assumed to index attentional selection (Mazza and Caramazza, 2011).

This coupling of the spatio-temporal coordinates of the sensory signal to a specific object representation enables identity integration between rapidly sampled content and slowly computed structure. Consequently, visual memory for an entire array of individual elements that requires binding of identity to location remains low throughout the integration bandwidth (**Figure 3** right panel; Wutz and Melcher, 2013). Consistent with the idea that individuation precedes identification, visual working memory performance rises gradually to asymptote under the influence of *backward masking* (Gegenfurtner and Sperling, 1993; Vogel et al., 2006). In contrast to the forward masking paradigm described above, backward masking is thought to reflect a disruption of processing after feedforward perceptual analysis is already completed (Scheerer, 1973a,b) but before consolidating information into visual working memory. This distinction in object processing stages between individuation and identification of objects is further fostered by task-specific activation patterns in parietal areas (Xu and Chun, 2006; Xu, 2007). Within such a “neural object-file” framework multiple visual objects are selected and individuated in an initial feedforward operation involving the inferior intra-parietal sulcus (IPS) and only subsequently identified and maintained in visual working memory (within superior IPS; Xu and Chun, 2009).

Step-wise, feedforward individuation of only a limited number of objects within a temporal buffer limits the temporal dynamics of vision. In real-time processing, however, delayed feedback systems (like the visual system; Felleman and Van Essen, 1991) exhibit asymptotic unstable behavior when confronted with signals with different latencies that have to be combined (Sandberg, 1963). Temporal buffering provides a solution to this problem by synchronizing convergent input streams. In this way, feedback processing, like identification, operates upon the outcome of the whole temporal buffer to ensure spatio-temporally coherent vision. This provides a possible solution to the problem of how to carve continuous sensory input into coherent objects, despite the presence of feedback loops. Temporal windows allow for the read-out of individual elements but also the integration of sensory flux into a dynamic stream of visual impressions (Öğmen, 1993; Wutz and Melcher, 2013).

## NEURAL MECHANISMS: ALPHA PHASE SYNCHRONIZES INDIVIDUATION AND INTEGRATION

It has been suggested that implementation of integration windows within perceptual processing might involve brain oscillations (Varela et al., 1981; Pöppel and Logothetis, 1986; Dehaene, 1993). Numerous studies have shown that the temporal relation between sensory stimuli and neural oscillations can alter the perceptual outcome. For example, psychophysical threshold estimates have been shown to vary with the phase of ongoing oscillatory activity (Busch et al., 2009; Mathewson et al., 2009) and recent evidence suggests even a causal link between the two (Neuling et al., 2012). Moreover, perceived simultaneity and sequentiality of apparent motion percepts depend on the phase of the occipital alpha rhythm (Varela et al., 1981; Gho and Varela, 1988). Such periodic fluctuations have previously been described as rhythmic background sampling of the sensory surrounding (VanRullen et al., 2007; Busch and VanRullen, 2010). These results suggest that oscillations impose a “perceptual frame” on feedforward processing such that integration and individuation of sensory signals depends on its periodic phase.

One key characteristic of brain oscillations is robust phase synchronization to transient input (Buzsáki and Draguhn, 2004). In addition to effects of ongoing oscillations prior to stimulus onset, stimulus evoked synchronization patterns might reveal how phase information influences perceptual integration. In this view, external stimulation results in a “reset” of functionally relevant oscillatory patterns such that their phase synchronization is locked to stimulus onset. Resets might in particular occur in response to transient sensory change, like saccadic eye movements or real-world transitions (i.e., stimulus onset). In fact, evoked responses to successfully detected and entirely missed stimuli differ extensively (Busch et al., 2009) and alpha phase-locking accounts for individual differences in a rapid visual discrimination task (Hanslmayr et al., 2005). Likewise, reset cyclic patterns in visual task performance have been reported in response to sudden flash events (Landau and Fries, 2012) or auditory sounds (Romei et al., 2012). Moreover, electro-cortical stimulation studies demonstrated a causal link between phase resets and perceptual performance by showing that repetitive transcranial magnetic stimulation (TMS) at 10 Hz synchronizes natural alpha oscillations (Thut et al., 2011) and biases spatial selection in visual tasks (Romei et al., 2010, 2011).

**FIGURE 4:**
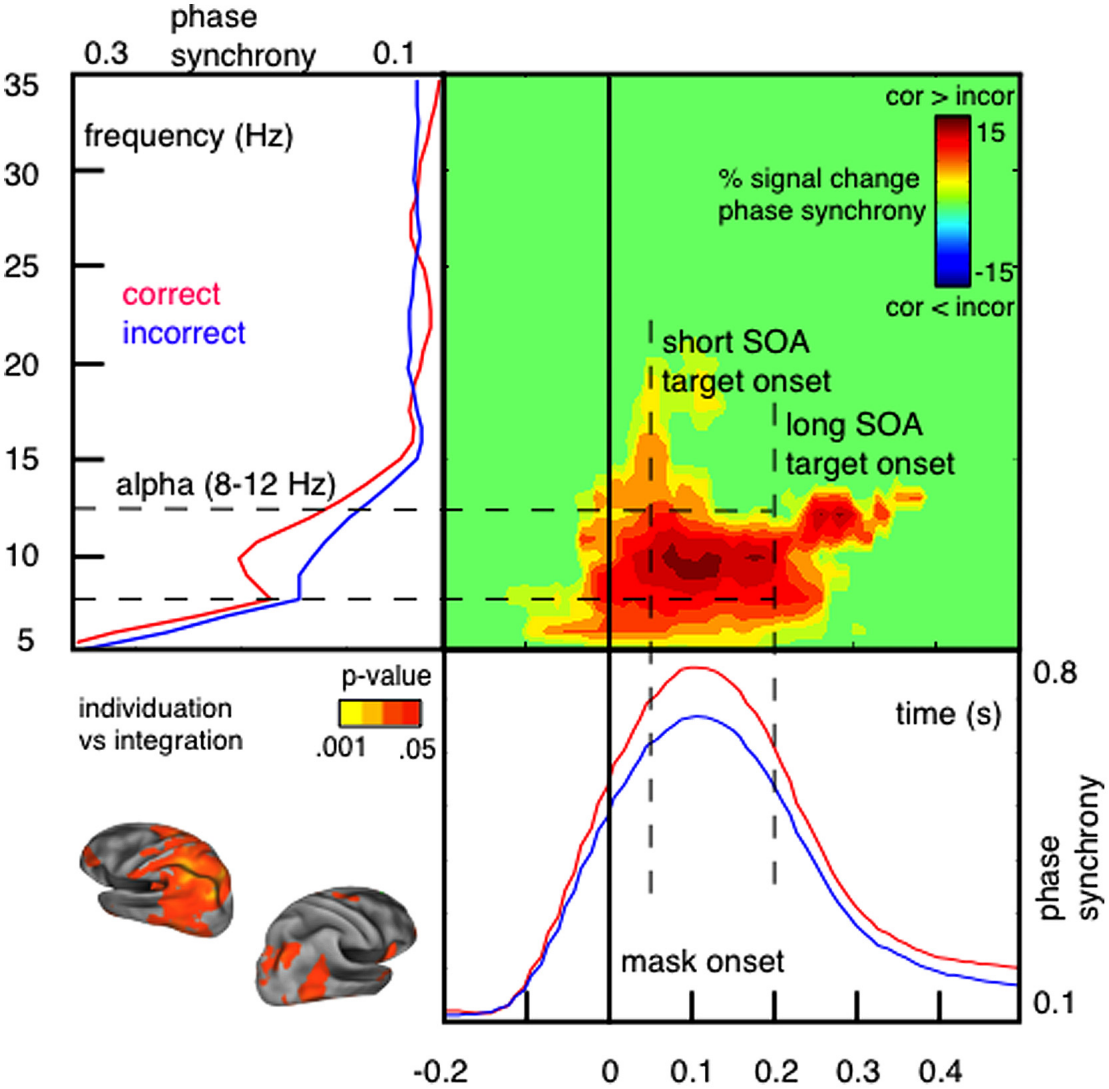
**Individuation and integration of visual signals depends on short-lived alpha phase synchrony shortly after stimulus onset.** Phase synchrony [measured with inter-trial coherence (ITC); Makeig et al., 2004; also called phase-locking factor (PLF); Tallon-Baudry et al., 1996] is higher within correctly individuated trials compared to incorrect integration. Phase synchronization in response to the masking sequence is short-lived (∼100 ms; lower panel) and selective for alpha oscillations (8–12 Hz; left panel). Neural generators of this effect are located in mostly left-hemispheric parietal areas (peak difference: left inferior parietal; localized using a linear constrained minimum variance (LCMV) beamformer algorithm; Van Veen et al., 1997). In particular, phase synchrony distinguishes between individuation and integration only for short SOA trials, in which temporal integration of rapid transients occurs; but not for long SOA trials, in which sensory changes exceed the critical integration time frame (Figure adapted with permission from Wutz et al., 2014).

In support for the idea of a link between phase synchronization and temporal integration windows, we have demonstrated that the perceptual outcome of integration masking depends on short-lived alpha phase synchrony over parietal sensors measured with MEG (Wutz et al., 2014). We contrasted trials in which observers accurately individuated low set-sizes of target items (up to 3) from masking persistence with trials in which mask and target elements integrated in time and individuation failed (see **Figure 2**). Correct individuation is accompanied by a reset selectively synchronizing alpha oscillations within a temporal window of around 100 ms (so for approximately one alpha cycle) shortly after onset of the masking sequence (**Figure 4**).

It is important to note that alpha phase synchrony reset by the masking sequence only distinguishes between individuation and integration of visual transients on rapid time scales (<100 ms; short SOA trials). Segregating sensory changes exceeding this critical time frame (long SOA trials) instead depends on slower beta power modulations prior to stimulus onset (Wutz et al., 2014). The time course of the alpha phase synchrony reset (≈100 ms; ≈one alpha cycle) is consistent with the perceptual effects of integration masking (Enns and Di Lollo, 2000; Breitmeyer and Öğmen, 2006; Wutz et al., 2012). These results suggest that short-lived alpha synchronization is in particular key for perceptual processing of fast sensory changes. Precise phase coding within this integration cycle (through e.g., eigenfrequency damped oscillations; Buzsáki and Draguhn, 2004) in response to sensory transitions might balance individuation of perceptual elements and integration of sensory flux to guarantee spatio-temporal coherent perceptual outcomes.

## IMPLICATIONS AND FUTURE DIRECTIONS

### THE MAGIC WINDOW: TIME AND CAPACITY LIMITS

Following Miller’s (1956) seminal paper discussing the “magic number” of 5–7 objects, the nature of these capacity limits has been a matter of intensive debate. Although a review of this extensive literature is beyond the scope here (for review see Cowan, 2000), it is important to note that the role of *time* in capacity limits has been almost neglected in any of the major theories. As described above, limits in the capacity of object individuation can be explained by the limited duration of visual persistence and the cycle of feedforward and feedback processing: in other words, temporal, rather than spatial, bandwidth. One advantage of a temporal window explanation of capacity is that capacity limits emerge naturally out of the rate of object individuation within this window of persistence, without the need to posit any *ad hoc* mechanisms.

In terms of neural implementations, the MEG evidence reported here, as well as related neuroimaging studies (Todd and Marois, 2004; Knops et al., 2014) suggest that neurons in posterior parietal cortex (PPC) may be involved in the individuation of objects. Specifically, capacity limits may reflect the spatial and temporal nature of attentional priority (saliency) maps in PPC (Melcher and Piazza, 2011; Franconeri et al., 2013; Knops et al., 2014). Unlike the priority maps in early visual areas (Zhang et al., 2012), attention priority maps in parietal cortex are thought to integrate bottom-up and top-down saliency estimates for objects over time (Bogler et al., 2011), allowing for object information to be accumulated and maintained (Mirpour et al., 2009; van Koningsbruggen et al., 2010). The results reviewed here emphasize the temporal aspects of the individuation process in determining attentional priority and capacity.

### INTERACTION WITH NATURAL VISION: RETINOTOPY AND VISUAL STABILITY

A fundamental challenge for the perception of coherent spatiotemporal objects is that objects move and so do our sensory receptors. In retinotopic space, object motion would be expected to create smear within the image plane along the motion path and blurry object representations (the so-called “moving ghost problem”; Öğmen and Herzog, 2010). Whereas motion smear can be reduced by mechanisms similar to meta-contrast masking (Chen et al., 1995; Purushothaman et al., 1998), the read-out of moving objects would still result in fuzzy perceptual form computations. In order to avoid such “ghost-like” appearances the visual system might rely on motion segmentation when computing non-retinotopic representations (Öğmen and Herzog, 2010). This development of non-retinotopic representations necessitates integration over a temporal interval on the order of 100–150 ms (Öğmen et al., 2006; Öğmen and Herzog, 2010; Otto et al., 2010). Temporal integration of feature persistence over this temporal interval has also been implicated in the use of spatial cues for motion direction in natural images (a “motion streak”; Geisler, 1999). In general, perceptual mechanisms responsible for motion and clear, un-smeared objects share functional characteristics and are capable of analyzing form and motion concurrently (Ramachandran et al., 1974; Burr, 1980; Burr et al., 1986), fostering the close link between object form and motion perception, and temporal integration over an interval of ca. 100 ms of image persistence.

The temporal window of individuation reviewed here might serve as a buffer to translate fast retinotopic representations into stable, but slower non-retinotopic (including spatiotopic, frame-based or object-based: Melcher, 2008; Lin and He, 2012) representations that are of particular importance when objects move or change quickly. Perceiving an object as an individual within a crowded scene requires the observer to represent an object’s spatiotemporal coordinates distinct from the background and from other individuals in the image. Such a structured perceptual representation contains information about sensory input that is invariant to its absolute retinotopic coordinates and gives rise to non-retinotopic form. Static input remains long enough on a well-defined location in the image, so that its associated features can be firmly attached to this location and capacity limits arise as a function of individuated locations within the image persistence. In case of fast changes in the image plane, however, only a subset of locations can be selected and individuated into non-retinotopic representations. In this way the need for higher temporal resolution balances with limits in the computation of stable non-retinotopic individuals in each single instance. Such an equilibrium might be essential in mediating between stable object and dynamic motion perception with minimal motion smear in the image plane.

Likewise, eye and head movements create a change in the retinal input and thus, potentially, a source of confusion when integrating information over time (for a discussion of the similarity between the effects of object and eye motion, see: Ağaoğlu et al., 2012). Typically, stable eye fixation periods last on the order of 150–300 ms in reading and natural viewing tasks (for review see Rayner, 1998). The external world seems stable despite these dramatic spatio-temporal disruptions in sensory information, perhaps relying on non-retinotopic object representations (Melcher and Colby, 2008; Burr and Morrone, 2011; Melcher, 2011).

We speculate that the visual system might deal with the problems of object and self-motion in a similar way, involving at least two stages of processing (see also Otto et al., 2010). At the first stage, relatively brief visual integration windows, such as those in visual masking studied here, combine information in a retinotopic manner over a time course that allows for feedforward processing. This time window is used to successively individuate spatio-temporal elements and hence stabilize sensory input. It is not coincidental, then, that the most brief eye fixations found in reading and natural viewing and intermediate-level visual integration windows would be of similar minimum durations since the goal of each new fixation is to sample part of the visual scene in order to individuate the most relevant objects. It would not make sense to move the eye before all of the information is sampled up to the level of object individuation, or to “mis-align” this integration window so that the saccade occurs right in the middle (integrating information during individuation from two different spatial locations). Moreover, the complete cycle of feedforward and feedback processing would tend to exhaust all of the useful information available from the fovea, making long fixation durations inefficient unless the information of the retina was dynamic or difficult to resolve.

At the second stage, however, information about the same object should be combined over time, over a longer time window and a non-retinotopic spatial reference frame. Accurate perception of object motion relies on non-retinotopic form computation (Öğmen et al., 2006). Likewise, there are a growing number of examples of spatiotopic perceptual effects across eye movements (for review, see Melcher and Colby, 2008; Burr and Morrone, 2011; Melcher, 2011) and there is converging evidence that this involves time scales of several hundred milliseconds (Zimmermann et al., 2013a,b). Overall, these studies suggest that there are both relatively brief, retinotopic integration windows and longer, spatiotopic windows.

One clear hypothesis from this idea is that retinotopic temporal integration windows should be reset by saccades and aligned to new eye fixations. As described above, it would be problematic if the basic object individuation process combined information from different spatial locations due to a saccadic eye movement changing retinal position during the integration window. Some evidence for a reset in the window of object individuation comes from studies of masking. Visual persistence, as measured by the missing dot task (Di Lollo, 1980), does not continue across saccades (Bridgeman and Mayer, 1983; Jonides et al., 1983) and masking can be disrupted by the intention to make a saccade (De Pisapia et al., 2010). On the other hand, the much longer temporal integration windows involved in apparent motion, over 100s of milliseconds, do not seem to be disrupted by saccades (Fracasso et al., 2010; Melcher and Fracasso, 2012). Further studies are needed to precisely define the relationship between fixation onset and the temporal windows of object individuation. The exact timing of temporal integration windows relative to eye movements might play a critical role for the impression of visual stability on rapid time scales. Such fast, feedforward computations might still involve retinotopic coordinates and therefore require saccadic remapping. However, much of the impression of visual stability might involve longer time windows that are not entirely retinotopic and thus do not require saccadic remapping.

### NEURAL SYNCHRONIZATION COORDINATES FEEDFORWARD AND FEEDBACK OBJECT PROCESSING

We have reported that the short-lived alpha phase synchronization reset by stimulus onset predicts perceptual performance on an integration task. Time- and frequency characteristics of this effect (100 ms at 10 Hz) point to an alpha phase reset involved in feedforward individuation of objects. This is in line with classical findings identifying partly reset alpha oscillations in event-related potential (ERP) signatures (especially in the N1 component, Makeig et al., 2002). The functional role of alpha oscillations in perception and cognition are debated. Recent advances, however, have associated alpha phase information with the selection and recognition of object representations (for review see Palva and Palva, 2007). In support of this view, but in contrast to spatial or numerical limits in object segmentation, we propose an account based on temporal bandwidth in which phase-locking couples external signals to alpha integration cycles. Processing limits might then arise as a result of feedforward encoding within one synchronized cycle. A temporal window model based on neural synchronization patterns has several interesting functional characteristics that could coordinate feedforward and feedback object processing.

Synchronous coupling to oscillatory dynamics can structure processing into cyclic time windows for coherent integration of convergent inputs that arrive with different latencies (Buzsáki and Draguhn, 2004). In this way alpha oscillatory cycles might reflect temporal reference frames as elementary building blocks in feedforward processing. In fact, alpha cycles have been previously discussed as segmenting input into discrete snapshots of ∼100 ms (VanRullen and Koch, 2003). In line with this view illusory motion reversals in the continuous wagon wheel illusion are most prominent at wheel-motion frequencies around 10 Hz and are correlated with alpha band amplitude in the ongoing EEG trace (VanRullen et al., 2005, 2006).

VanRullen and Koch (2003) also suggested a possible way to read-out object information within such a temporal window that might involve coupled networks of nested oscillatory sub-cycles (coding individual content) within slow-wave carriers (defining the temporal reference frame). Such neural networks are capable of representing individual information by means of frequency-division multiplexing (Lisman and Idiart, 1995). Especially, phase-amplitude coupling between α- and γ-frequency bands could prioritize the selection of multiple visual objects (Jensen et al., 2012, 2014). In this view the selection of individual items might be regulated via timed release of inhibition within one alpha cycle (Van Rullen and Thorpe, 2001; Klimesch et al., 2007; Jensen et al., 2012). Indeed, neural network dynamics of individuation can be modeled based on inhibition between competing items in a saliency map (Knops et al., 2014). Whereas multiplex coding is a well-established principle of neural function (O’Keefe and Recce, 1993; Kayser et al., 2009; Siegel et al., 2009), future work is needed to determine its functional significance for human visual cognition. Our results support the view that oscillatory synchronization might represent multiplexed phase coding and suggest that object capacity limits can arise, not only by the read-out speed of individual elements, but also from the bandwidth of the carrier function.

Importantly, integration windows can help to coordinate visual processing dynamically, because phase synchronization occurs in response to internal or external changes in input (via phase resetting; Buzsáki and Draguhn, 2004; Buzsáki, 2006; for review see Thut et al., 2012). In this way, brief phase synchronization might contribute to the rapid coordination of distributed neuronal populations (like the retinotopically organized areas along the visual hierarchy; von der Malsburg, 1981; von der Malsburg and Schneider, 1986; Singer and Gray, 1995; Fries, 2005). This might be important in order to cope with the combinatorial complexity of crowded visual scenes that contain individual elements that can consist of a nearly infinite number of feature combinations and can appear at any given moment in time or spatial location. This flexibility in combining arbitrarily complex features over space and time would seem to require neural network communication. In line with this view, phase synchronization has been hypothesized to sub-serve cross-modal integration or feature binding and to gate the information flow between local neuronal ensembles (Singer, 1999; Salinas and Sejnowski, 2001). Consistent with this idea, phase synchrony between distributed processing sites has been demonstrated to predispose visual perception (Hipp et al., 2011), route selective attention (Siegel et al., 2008; for review see Womelsdorf and Fries, 2007), predict individual working memory capacity (Palva et al., 2010) and reflect higher-level temporal processing limits (Gross et al., 2004).

Our results reveal wide spread synchronization patterns in parietal cortices locked to stimulus onset already at the level of object segmentation. We argue that vision makes use of phase synchronization as a temporal reference frame in which distributed processing can be orchestrated and aligned to input transitions. Reset synchronization patterns might therefore coordinate feedforward and feedback mechanisms involved in encoding complex and dynamic visual scenes with nearly real-time speeds. In this framework, temporal windows might reflect a neural strategy for coherent perception of objects in space and time.

## CONCLUSION

As described above, there is accumulating psychophysical and electrophysiological evidence for an intermediate-level temporal window involved in the individuation of a small number of relevant objects in a scene. Individuation capacity increases in steps within the lifetime of visual persistence of the stimulus, suggesting that visual capacity limitations arise as a result of the narrow temporal window of sensory persistence. In contrast to the main theories based on spatial slots or finite spatial resources, these findings suggest that *time* is the critical factor in the emergence of capacity limits. In this way, capacity limits can be seen as a result of the need of the visual system to coordinate feedforward and feedback processes. The cycle of feedforward and feedback processing reflects a compromise between the competing needs of a perceptual system to integrate information over extended periods of time (to get a better estimate of stable object and event properties) and sensitivity to changes in the environment.

## Conflict of Interest Statement

The authors declare that the research was conducted in the absence of any commercial or financial relationships that could be construed as a potential conflict of interest.
